# New Insights into the Structure of Kappa/Beta-Carrageenan: A Novel Potential Inhibitor of HIV-1

**DOI:** 10.3390/ijms222312905

**Published:** 2021-11-29

**Authors:** Irina Yermak, Stanislav Anastyuk, Anna Kravchenko, William Helbert, Valery Glazunov, Andrey Shulgin, Pavel Spirin, Vladimir Prassolov

**Affiliations:** 1 G.B. Elyakov Pacific Institute of Bioorganic Chemistry, Far Eastern Branch of the Russian Academy of Sciences, 100 Let Vladivostoku Prosp., 159, 690022 Vladivostok, Russia; mentat-vvo@yandex.ru (S.A.); kravchenko_25.89@mail.ru (A.K.); glazunov@piboc.dvo.ru (V.G.); 2 CERMAV, CNRS and Grenoble Alpes Université, BP53, CEDEX 09, 38000 Grenoble, France; william.helbert@cermav.cnrs.fr; 3 Engelhardt-Institute of Molecular Biology, Russian Academy of Sciences, Vavilova, 32, 119991 Moscow, Russia; ashu69@mail.ru (A.S.); discipline82@mail.ru (P.S.); prassolov45@mail.ru (V.P.); 4 Moscow Institute of Physics and Technology (State University), Institutskiy per., 9, Dolgoprudny, 141701 Moscow, Russia; 5 Center for Precision Genome Editing and Genetic Technologies for Biomedicine, Engelhardt Institute of Molecular Biology, Russian Academy of Sciences, Vavilova, 34/5, 119334 Moscow, Russia

**Keywords:** carrageenan, oligosaccharides, structure, HIV-1

## Abstract

New insights into the structure of the hybrid κ/β-carrageenan (κ/β-CRG) of the red alga *Tichocarpus crinitus* have been obtained. Carrageenan oligosaccharides were prepared through the chemical and enzymatic depolymerization of κ/β-CRG with κ-carrageenase and its the enzyme-resistant fraction. The composition and distribution of the repetition units of κ/β- CRG were investigated by using the negative ion tandem MALDI-TOFMS and ESIMS method, which made it possible to prove and characterize the hybrid structure of this polysaccharide. An analysis revealed the blockwise distribution of the long β-blocks along the polysaccharide chain, with the inclusion of κ/β, μ/ν-blocks and some ι-blocks. Furthermore, the desulfated κ/β-CRG was shown to contain of –G–D– repeating units up to 3.5 kDa. Previous studies have demonstrated that CRGs suppress the replication of several viruses. Here, we established that κ/β-CRG and its oligosaccharides significantly inhibit the transduction efficiency of replication-defective lentiviral particles pseudotyped with the envelope proteins of three different viruses. We found that the polysaccharide and its oligosaccharides strongly reduced the transduction efficiency of lentiviral particles pseudotyped with GP160—the envelope protein of the human immunodeficiency virus HIV-1—when added to T-lymphocyte Jurkat cells. The CRG oligosaccharides displayed significantly higher antiviral activity.

## 1. Introduction

Carrageenans (CRGs) are a family of water-soluble linear sulfated galactans extracted from red algae. They are appreciated for their structural diversity, which is associated with a large panel of physico-chemical properties and biological activities. They feature a primary backbone structure based on alternating three-linked β-D-galactopyranose and four-linked α-D-galactopyranose residues, and several types of these polysaccharides are identified on the basis of the structure of the disaccharide repeating units, by the sulphation pattern, and by the presence of 3,6-anhydrogalactose (A unit) as a four-linked residue [1,2]. To describe the complex CRG structure, a uniform letter code nomenclature has been developed [1]. Based on this nomenclature, the three-linked β-D-galactopyranose is the **G** unit, while four-linked α-D-galactopyranose D-unit or DA-units, respectively.

The most common types of CRGs are named κ-, ι-, and λ-CRGs, based on the structure of the major disaccharide repeating units. Therefore, the terms κ- and ι-CRGs are used for polysaccharides that contain κ-carrabiose (G4S-DA) and ι-carrabiose moieties (G4S-DA2S), respectively, as the main components. A new type of CRG, in which the three-linked residues are not sulphated and the four-linked units lack sulphate at C-2, was first reported by Greer and Yaphe [3]. This type was called β-CRG, or furcelaran, since it was extracted from *Furcellaria lumbricalis*. Later, other researchers demonstrated that ion-independent β-CRG may also be isolated from *Eucheuma speciosa* and *Endocladia muricatum* [4]. Natural CRGs are often hybrids of more than one of these units and are made of several carrabiose moieties [5,6]. Several carrabiose combinations have been demonstrated, such as κ/β- (DA-G4S/DA-G) CRG, which is found in *F. lumbricalis* and *Euchema gelatinae* [7,8].

The red alga *Tichocarpus crinitus* is a unique specimen of the family Tichocarpaceae that is widely spread in the seas of the Far East. In a previous study, we demonstrated that the gelling polysaccharides isolated from the vegetative forms of *T. crinitus* belonged to the κ/β-carrageenans type (DA-G4S/DA-G), with predomination (80%) of the carrabiose units of the κ-type [9]. Later, we carried out an analysis of the structural heterogeneity of κ/β-CRG from *T. crinitus* digested with κ-carrageenase from marine bacterium *Pseudoalteromonas carrageenovora* [10]. The application of tandem ESIMS allowed the fast estimation of the compositional and structural features of the oligosaccharide mixtures, obtained from the κ/β-CRG by different methods of degradation. According to our results, the electrospray ionization tandem mass spectrometric (ESIMS/MS) investigation of the oligosaccharide products obtained by recombinant κ-carrageenase digestion of κ/β-CRG of *T. crinitus* revealed that the mixture contained κ-carrabiose as its main component, κ-carratetraose, and a set of hybrid oligosaccharides. Using instrumental techniques and gel-permeation chromatography, the composition and distribution of the carrabiose units in the mixture of oligosaccharides was estimated [11]. However, the composition of the enzyme-resistant fraction of κ/β-CRG remained unexplored. Through the complete structural analysis of oligosaccharides obtained by acid hydrolysis of CRG resistant fraction combined with tandem MALDI-TOFMS, it is possible to determine the composition and the distribution patterns of the carrabiose moieties along the polysaccharide chains and to more accurately characterize the complex structure of the hybrid CRG.

In our previous study, we established that κ/β-CRG obtained from algae *T. crinitus* possesses antiviral activity against Tobacco mosaic virus (TMV). Our observation indicated that the κ/β-CRG inhibited TMV infection in detached tobacco leaves at early stages [12]. These data made it possible to consider it as a potential antiviral plant protectant. Different types of CRGs have been found to be active against a variety of viruses, including metapneumovirus [13], rabies [14], chlamydial infection [15], and human papillomavirus (HPV) [16], as well as a range of sexually transmitted HPV types [17]. Usually, the antiviral activity of polysaccharides correlates with their molecular weight (Mw). The higher the average molecular weight (MW), the higher the antiviral activity for many cases [18]. At the same time, low-molecular-weight derivatives of κ-CRG with a MW of 3.5 and 10 kDa were obtained. These oligosaccharides exhibited high antiviral activity and were proposed as candidates for the creation of a drug against influenza [19]. At the same time, the antiviral activities of κ- and κ/β-CRGs were higher than those of oligosaccharides, as we demonstrated previously [20].

The use of infectious viruses greatly complicates the search and testing of antiviral drugs. Replication-defective lentiviral viruses are widely used to study the mechanisms of viral infection and to test potential antiviral drugs [PMID: 19118579, PMID: 23286882, PMID: 32466195]. Furthermore, retrovirus-based systems were successfully used to investigate the activity of potential inhibitors that suppress the viral infection by blocking the initial contact of viral particle with the target cell (PMID: 21935898, PMID: 33330839, PMID: 23966033).

The aim of this study was to determine the structural properties of the enzyme-resistant part of hybrid κ/β-CRG from *T. crinitus* by mass spectrometric analysis of oligosaccharides derived through mild acid hydrolysis and to determine the antiviral activity of κ/β-CRG and its oligosaccharide using the model viral system, based on replication-defective lentiviruses (RDLVs).

To assess the antiviral activity of CRG and its oligosaccharides, we used a set of RDLVs carrying marker gene encoding green fluorescent protein (GFP). We found that κ/β-CRG caused a strong antiviral action when added in non-toxic concentrations. Furthermore, using the RDLVs pseudotyped with different envelope proteins, we found that the antiviral effect of CRGs depends on the proteins used for coating, which suggests the possible specific target for its antiviral activity.

## 2. Results and Discussion

### 2.1. Carrageenan and Oligosaccharides

The polysaccharide was extracted from *T. crinitus*, purified from low-molecular-weight impurities, and fractionated with potassium chloride into the gelling (KCl-insoluble) and non-gelling (KCl-soluble) fractions. The structure of the gelling polysaccharide was studied by ^13^C nuclear magnetic resonance (NMR) and Fourier-transform infrared spectroscopy (FT-IR), and the obtained spectra were compared with the spectra of polysaccharides isolated from *T. crinitus* earlier by our group [9]. According to data obtained by spectroscopy, the KCl-insoluble polysaccharide from *T. crinitus* featured a hybrid κ/β-CRG structure. The viscometric molecular weight of κ/β-CRG, calculated by the Mark-Kuhn–Houwink equation, was 350 kDa (Table 1).

The oligosaccharides were obtained through mild acid hydrolysis of κ/β-CRG and the experimental conditions applied were suitable for the preparation of oligosaccharides of κ/β-CRG, since the content of sulfate ester groups and DA residues were conserved (Table 1), in agreement with previous investigations [20].

These observations were confirmed by the recorded FT-IR spectra (Supplementary S1). The FT-IR spectra of the LMW–CRG as well as the κ/β-CRG contained characteristic absorption bands for κ/β-CRG [9,21]. Notably, an intense absorption band in the region of 1258/1230 cm^−1^ indicated the presence of a significant number of sulfate groups (–S = O asymmetric vibration) that was in agreement with results of the chemical analysis. The absorption bands at 929 and 848 cm^−1^ in the FT-IR spectra of the polysaccharides were characteristic of 3,6-anhydrogalactose (C–O vibration) and the secondary axial sulphate group at C-4 of the 3-linked β-D-galactose residue, respectively. This made it possible to assign the polysaccharides to κ-CRG. Moreover, the absorption band at 892 cm^−1^ in the FT-IR spectrum of the polysaccharide and its oligosaccharide also indicated the presence of non-sulphated β-D-galactose residues, typical of β-CRG (Supplementary S1).

The molecular weight of the LMW product calculated using the reaction of reducing sugars with ferricyanide was 2.1 kDa. Commercial κ-CRG from Sigma-Aldrich was also used (CRG-S).

### 2.2. The Enzyme-Resistant Fraction of κ/β-Carrageenan RF/CRG

Recently, instrumental methods (mass spectrometry together with NMR spectroscopy) were applied to the analysis of the mixture of oligosaccharides released from κ/β-CRG of *T. crinitus* by enzymatic digestion with κ-carrageenase from marine bacterium *P. carrageenovora.* It was shown that the mixture of oligosaccharides (degree of polymerization DP up to 20) produced by this enzyme contained κ-carrabiose and κ-carratetraose as the major components. Furthermore, a set of hybrid κ/β oligosaccharides was detected at lesser abundance and along with minor components. It was also found that some percentage (~20%) of the polysaccharide was not hydrolyzed by the enzyme–the resistant fraction (RF/CRG) and thus eluted in a mixture as high molecular weight fraction (MW = 160 kDa, [11]) in size exclusion chromatography. To clarify the κ/β-CRG-structure, oligosaccharides of this RF/CRG- fraction were obtained.

### 2.3. Mass Spectrometry of Oligosaccharides Obtained by Mild Acid Hydrolysis of RF/CRG

**Figure 1:**
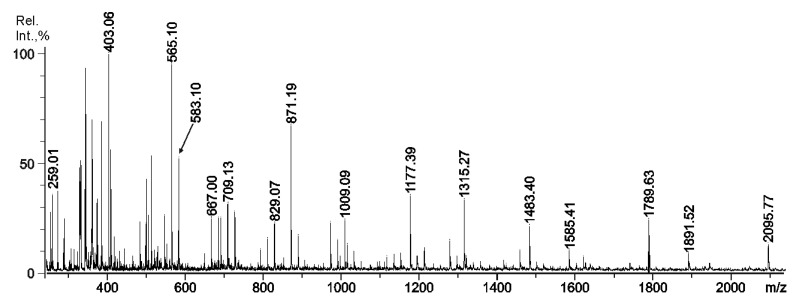
Negative-ion MALDI-TOFMS of the oligosaccharide mixture obtained by acid hydrolysis from RF/CRG polysaccharide.

Mild acid hydrolysis of RF/CRG was carried out at 37 °C (Section 3.3), and the structural features of the oligosaccharides were investigated by MALDI-TOFMS and ESIMS/MS techniques. According to the data obtained in the negative-ion mode (Figure 1), the mixture of hydrolysis products contained a set of oligosaccharides up to ~2 kDa. The structural features of some interesting components were investigated by the ESIMS/MS technique, rather by the MALDI tandem technique, because the ESIMS instrument was able to isolate the precursor ion more accurately and allowed the precise control of the collision energy during the collisionally-induced dissociation (CID) process. The suggested content/structural features of the oligosaccharides found in the mixture are collected in Table 2. Some of the ions were suspected to be fragment ions by comparison of the MALDI and ESIMS spectra. Obviously, one cannot exclude that the oligosaccharides observed by ESIMS were not fragments themselves. Notably, some fragments of RF/CRG, along with fragments of κ/β-blocks (*m*/*z* 565.1), were parts of ι-blocks (*m*/*z* 667.0), which were also found by the ^1^H NMR technique in the products of enzymatic degradation earlier [11]. The oligosaccharides containing D6S (underlined in Table 2), which were the precursors for building blocks of β-chains (*m*/*z* 685.1), were also observed.

**Figure 2:**
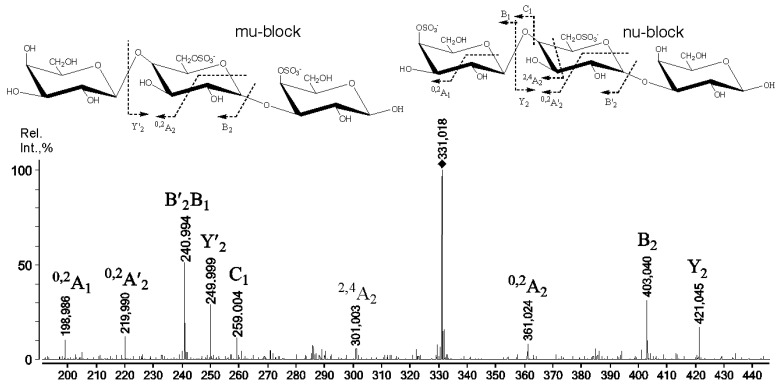
Negative-ion tandem ESIMS of ion at *m*/*z* 331.018 obtained through mild acid hydrolysis from RF/CRG.

The negative-ion tandem ESIMS of the ion at *m*/*z* 331.018 (Figure 2) contained D6S residues. This was confirmed by the observation of a characteristic ^0,2^X/^0,2^A-type fragment ions from the hexapyranose opening. It must be noted that the mechanism of the aforementioned ion–molecular reaction involved the proton at the hydroxyl group of C-3 [22]. This allowed us to precisely determine the exact position of the four-linked sulphated at the C-6 galactose (D6S) residue by the ESIMS/MS technique (Table 2).

**Figure 3:**
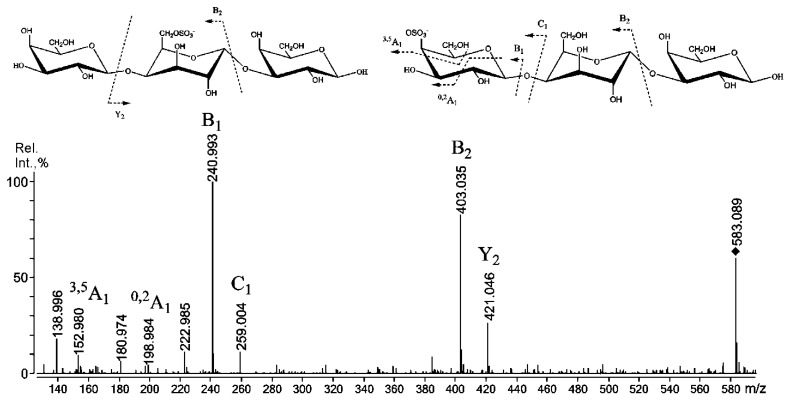
Negative-ion tandem ESIMS of ion at *m*/*z* 583.089, obtained through mild acid hydrolysis from RF-CRG.

Additional negative-ion tandem ESIMS of the hexose-containing trisaccharide ion at *m*/*z* 583.089 also exhibited A-type fragment ions, which are rarely observable in the MS of carrageenan-derived oligosaccharides [23]. A ^3,5^A_1_-type fragment ion at *m*/*z* 152.980 and a ^0,2^A_1_-type fragment ion at *m*/*z* 198.984 suggested that the hexose residue in the structural variant of the ion under study (Figure 3, right side) was sulphated in position C-4 [24].

**Figure 4:**
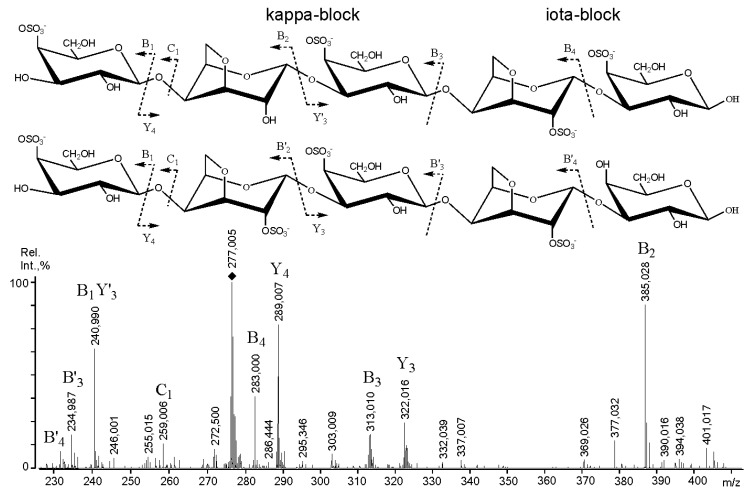
Negative-ion tandem ESIMS of ion at *m*/*z* 277.005, obtained through mild acid hydrolysis from *RF-CRG.*

The most complex negative-ion tandem ESIMS of the ion at *m*/*z* 277.005, which contained four sulphate groups per molecule, also demonstrated (Figure 4) that at least two structural variants of the ion under study simultaneously existed in the mixture. The interpretation of the ESIMS was typical [10] and was reduced to the correct arrangement of the Y- and B-type ions according to their charge state. The ion under study was built of κ-blocks with ι-block insertion since DA2S residues were detected. One could not exclude the possibility that the selected fragment was actually a whole ι-block; however, due to mild acid hydrolysis, it was partially desulphated. Again, the presence of ι-blocks in RF/CRG at detectable 1^3^C NMR levels was demonstrated in a previous study [11].

**Figure 5:**
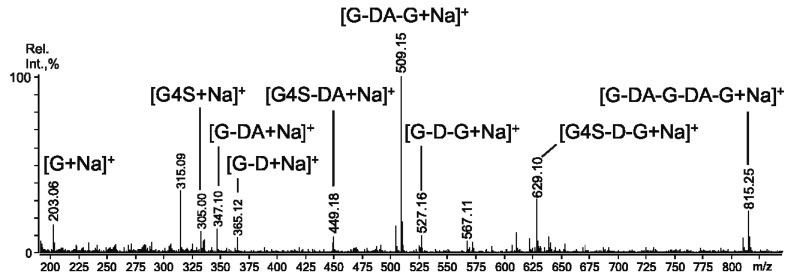
Positive-ion ESIMS of the oligosaccharide mixture, obtained through acid hydrolysis from RF/CRG polysaccharide.

The positive-ion ESIMS of oligosaccharides derived from RF/CRG by mild acid hydrolysis (Figure 5) revealed important information on the composition of the fraction under study. The following series of ions corresponds to unsulphated (probably loss of a sulphate from C-6) blocks of predecessors of β-units: *m*/*z* 203.06 ([G +Na]^+^), *m*/*z* 365.12 ([G-D +Na]^+^), and *m*/*z* 527.16 ([G-D-G +Na]^+^). The ion signals at *m*/*z* 509.15 ([G-DA-G +Na]^+^) and *m*/*z* 815.25 ([G-DA-G-DA-G +Na]^+^) exhibited the highest intensity. This suggests that the unsulphated β-units were rather extensive. Being unsulphated, the cleavage rate of these fragments was higher under acid hydrolysis conditions [25].

However, to examine the incorporation of precursors (μ- and ν-blocks) that were detected by negative-ion ESIMS/MS (see above), we submitted the native κ/β-CRG sample to partial degradation under solvolytic desulphation conditions (Supplementary S2), which were successfully employed previously to obtain information on the hexose-based core/impurities of more labile sulphated polysaccharides: fucoidans [26]. In brief, the MALDI-TOFMS of the mixture obtained after solvolysis (Supplementary S3) demonstrated the presence of polysaccharide ion distribution from 1.5 to 3.5 kDa, with a maximum at ~2.5 kDa. The mass difference between the ion peaks was 162 Da; thus, a polysaccharide was represented by chains built up of hexose residues. The ^13^C NMR analysis (see Supplementary S2) of this degraded polysaccharide clearly demonstrated that it was made of →G→D→ chains. The sulphated variants were detected by negative-ion tandem mass spectrometry as fragments of μ- and ν-blocks (Figure 3) in the mild acid hydrolysate of resistant fraction κ/β-CRG. The relatively large amount of these structural motifs in the RF/CRG fraction can be observed in Figure 4. This observation is in agreement with the results of previous work on *T. crinitus*, where it was shown that CRG exhibited poor rheological properties, while other hybrid κ/β-CRG from *F. lumbricalis* exhibited good gelling properties. It was suggested that the CRG under study probably had block-wise distribution of κ- and β-blocks along the polysaccharide chain, while the polysaccharide from *F. lumbricalis* featured random distribution of κ- and β-carrabiose, which led to a more regular distribution of charges [11].

Thus, using instrumental methods, we revealed that the κ/β-carrageenan from the red alga *T. crinitus* featured extensive (up to 3.5 kDa) →G→D→ chains of the predecessors (μ- and ν-blocks) and extensive chains of β-blocks.

### 2.4. Antiviral Activity of κ/β-CRG and Its Oligosaccharides

**Figure 6:**
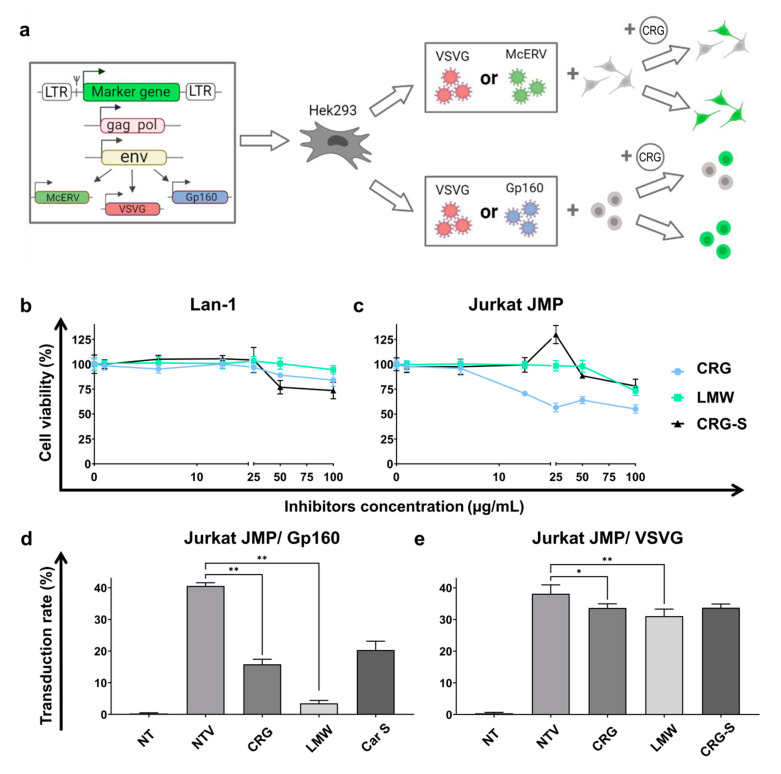

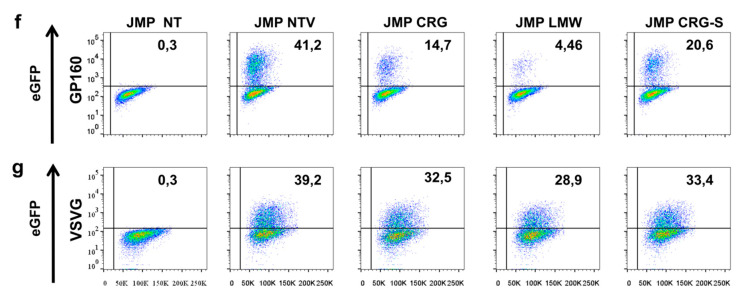
Antiviral action of κ/β-CRG and their oligosaccharide (LMW) against replication defective viruses pseudotyped with HIV-1 GP160 or VSVG envelope proteins. (**a**) Scheme of the experiment. The recombinant plasmid LeGO G2 encoding marker protein GFP, plasmid encoding structural proteins (gag-pol) and one of three plasmids encoding envelope proteins (env) were used for generation of RDLVs, pseudotyped with HIV-1 envelope protein GP160, vesicular stomatitis virus VSVG or endogenous retrovirus McERV. Jurkat JMP (spherical) or Lan-1 (fibroblast-like) cells were treated with CRG and used for trunsduction. The percentage of transduced cells (colored with green) was analyzed by flow cytometry (**b**) The viability of Lan-1 cells treated with CRG, LMW and CRG-S in concentration range up to 100µg/mL. (**c**) The viability of Jurkat JMP cells treated with CRG, LMW and CRG-S in a concentration range up to 100µg/mL. (**d**) Percentage of GFP-positive Jurkat JMP cells measured by flow cytometry 72 h after adding supernatans containing GP160-pseudotyped RDLVs at presence of CRG (κ/β-CRG, LMW, Car-S). NT: non-transduced cells and not treated with CRGs, NTV: transduced cells but not treated with CRGs were used as controls. (**e**) Percentage of GFP-positive cells measured by flow cytometry 72 h after adding supernatants containing VSVG-pseudotyped RDLVs at presence of CRGs. (**f**) Flow cytometry analysis of Jurkat JMP (JMP) cells transduced with RDLV-GP160 and treated with CRGs. (**g**) Flow cytometry analysis of Jurkat JMP (JMP) cells transduced with RDLV-VSVG and treated with CRGs. * *p* < 0.05, ** *p* < 0.005 as calculated by Mann–Whitney non-parametric test.

Next, to evaluate the antiviral activity of CRG, we used a model system based on RDLVs. For this, a set of replication-defective viruses bearing a marker gene encoding enhanced green fluorescent protein (eGFP) was used (Figure 6a). Three types of RDLV-pseudo-HIV-1 particles pseudotyped with envelope proteins of various viruses were obtained: Lenti-eGFP-VSVG (RDLV-VSVG) (pseudotyped with an envelope protein of the vesicular stomatitis virus VSVG), Lenti-eGFP-GP160 (RDLV-VSVG) (pseudotyped with the envelope protein of the human immunodeficiency virus HIV-1), and Lenti-eGFP-McERV (RDLV-McERV) (pseudotyped with an envelope protein of the endogenous retrovirus *Mus caroli*–McERV). To evade the toxic effect of CRGs on cells, the minimal non-toxic concentrations were determined for Lan-1 and Jurkat JMP and cells and used in further experiments (Figure 6b,c).

To evaluate the antiviral activity of CRGs, we used several combinations of RDLVs and cell lines: (1) RDLV-VSVG and RDLV-GP160 for infection and CD4+ Jurkat JMP (PMID: 23966033) cells as a target; (2) RDLV-VSVG and RDLV-VSVG for infection and Lan-1 cells as a target. The percentage of infected cells (transduced with RDLVs) treated with CRGs was quantitatively assessed by flow cytometry and compared to cells not treated with CRGs but transduced with RDLVs (NTV) and cells not treated with carrageenans and not transduced with RDLSs (NT) (Figure 6d,e). The typical scatter plots obtained with the flow cytometry assay (Figure 6f,g) demonstrate the distribution of the cells treated with CRGs and transduced with RDLVs. The data demonstrate that CRGs block the entry of RDLVs into the cells. The volumes of virus-containing medium were used to achieve a transduction efficiency of no more than 40%.

We found that the treatment of infected cells with κ/β-CRG and their oligosaccharides (LMW) caused a significant reduction in the proportion of eGFP positive cells compared to the control sample (NTV) (Figure 6d,e). Interestingly, oligosaccharides were found to be significantly more efficient at suppressing the infection. Treatment with LMW-CRG at a concentration of 25 µg/mL led to a tenfold reduction in the infection efficiency of lentivirus pseudotyped with HIV-1 envelope protein gp160 (Figure 6d,f). κ/β-CRG at a concentration of 5 µg/mL was also found to be very efficient at suppressing HIV-1 infection. Next, we compared the antiviral potential of κ/β-CRG and their oligosaccharides (LMW) with commercial Car-S (10 µg/mL) obtained from Sigma-Aldrich (Merck KGaA, Germany) and established that CRG and Car-S exert mostly comparable effects on infection efficiency opposite to LMW, which was found to be significantly more efficient than Car-S. Interestingly, the treatment with CRGs of cells infected with replication-defective lentivirus pseudotyped with VSVG does not cause so pronounced antiviral action. The most significant antiviral effect was evaluated when the cells were treated with LMW, which caused a reduction of the number of infected cells of up to 25% in the whole population of transduced cells compared to the control sample not treated with CRGs (Figure 6e,g).

**Figure 7:**
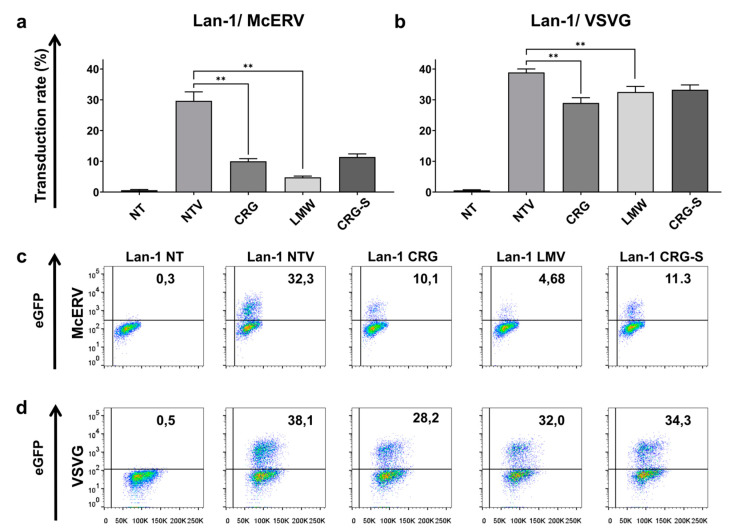
Antiviral action of κ/β-CRG and their oligosaccharide against replication defective viruses pseudotyped with McERV or VSVG envelope proteins. (**a**) Percentage of GFP positive Lan-1 cells measured by flow cytometry 72 h after adding supernatans containing McERV-pseudotyped RDLVs at presence of carrageenans (κ/β-CRG, LMW, Car-S). NT: non-transduced cells and not treated with CRGs, NTV: transduced cells not treated with CRGs were used as controls. (**b**) Percentage of GFP positive cells measured by flow cytometry 72 h after adding supernatants containing VSVG-pseudotyped RDLVs at presence of CRGs. (**c**) Flow cytometry analysis of Lan-1 cells transduced with RDLV-McERV and treated with CRGs. (**d**) Flow cytometry analysis of Lan-1 cells transduced with RDLV-VSVG and treated with CRGs. ** *p < 0.005* as calculated by Mann–Whitney non-parametric test.

Next, we used Lan-1 adhesive cells of neuronal origin to evaluate whether antiviral CRGs are restricted to GP160 pseudotyped cells and whether the cell origin may impact the antiviral action of the CRGs. For that, we used RDLV-McERV and RDLV-VSVG. The McERV envelope protein was isolated earlier from endogenous retrovirus of wild species *Mus caroli* (PMID: 18463156). The McERV virus uses proteolipid plasmolipin as an entry receptor; therefore, we used plasmolipin-positive Lan-1 cells as targets for transduction with McERV-pseudotyped RDLVs. We found that treatment with CRGs caused a significant reduction in the percentage of eGFP-positive cells infected with replication-defective virus pseudotyped with the McERV envelope protein. As with GP160-pseudotyped lentiviruses and Jurkat JMP cells, the most pronounced antiviral action caused CRGs oligosaccharides LMW (Figure 7a), reducing the percentage of infected cells up to sevenfold.

We evaluated that LMW was not so effective at suppressing the infection caused by RDLVs pseudotyped with VSVG. A slight but significant suppression was achieved when κ/β-CRG was added, reducing the percentage of infected cells up to 25% compared to the control cells not treated with CRGs (Figure 7b). Importantly, κ/β-CRG and commercial Sigma CRG (Car-S) (all taken at a non-toxic concentration of 25 µg/mL) were also found to perform a strong antiviral action when Lan-1 neuronal cells were infected, reducing the percentage of infected cells up to threefold (Figure 7a,c). Notably, κ/β-CRG and its oligosaccharide mostly prevented the infection of target cells that followed out of the scatter-plots, representing a significant decrease in the percentage of infected cell but not the simple suppression of fluorescence intensity (Figure 7c,d). The same action of the CRGs was also found when RDLVs, pseudotyped with the HIV-1 envelope protein GP160, were used for transduction (Figure 6f).

**Figure 8:**
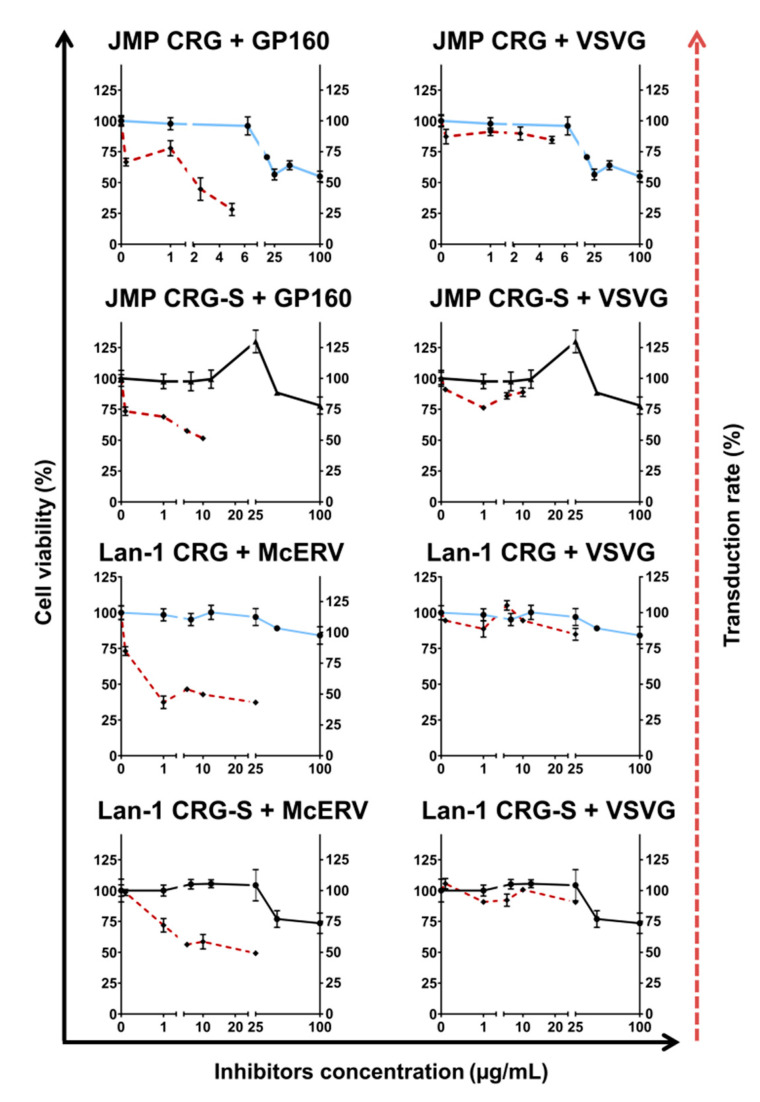
The antiviral action of κ/β-CRG compared to its cytotoxic activity. The solid lines represent the percentage of viable cells Jurkat JMP or Lan-1 (left *Y*-axis) treated with CRGs (κ/β-CRG or Car-S) in concentration range starting from 0.01 and up to 100 µg/mL (*X*-axis). The amount of living cells treated with CRGs was calculated and represented as a percentage to untreated cells. The dotted lines represent the percentage of transduced cells (right dotted transduction rate *Y*-axis) when RDLVs containing medium was added.

To demonstrate the relationship between the dose of carrageenan added to cells and their antiviral action, we used several concentrations of CRGs and found that the antiviral effect of CRGs started from the minimal nanomolar concentrations when the cells were transduced with GP160 or VSVG-pseudotyped RDLVs (Figure 8).

The addition of CRGs to infected cells at a concentration of 1 µg/mL caused a significant reduction in the percentage of infected cells, which became more pronounced when the CRGs were added at higher concentrations. The slight toxic effect of CRG on Jurkat JMP cells started at approximately 10 µg/mL. Importantly, CRG was not shown to be toxic up to a concentration of 100 µg/mL when added to Lan-1 cells of neuronal origin.

## 3. Materials and Methods

### 3.1. Algae

The red algae *T. crinitus* (Tichocarpaceae) were harvested at the Peter the Great Bay, Sea of Japan, and identified based on their morphological and anatomical characteristics by Prof. E. Titlyanov and T. Titlyanova (*National Scientific Center of Marine Biology, Far-Eastern Branch of the Russian Academy of Sciences*), using a transmission electron microscope. The selected seaweeds were in vegetative form, lacking any reproductive organs. The bleaching of the seaweed was obtained by maintaining the specimen in pure acetone for 3 days prior to drying it in air. The algae were washed with tap water to remove excess salt.

### 3.2. Extraction of Carrageenan

Dried and milled algae (50 g) were suspended in hot water (1.5 L) and the polysaccharides were extracted three times at 80 °C for 3 h in a boiling water, filtered through a Vivaflow200 membrane (Sartorius, Germany) with a pore size of 100 kDa, concentrated and precipitated polysaccharides with a triple volume of 96% ethanol, as described in previous research [9]. Next, the polysaccharides were separated into gelling (KCl-insoluble) and non-gelling (KC1-soluble) fractions, as described previously. The structure of the gelling polysaccharide was established according to the published protocol [9]. Gelling KCl-insoluble fraction as κ/β-CRG was used in investigation.

### 3.3. Preparation of Oligosaccharides from the κ/β-CRG

The oligosaccharides were obtained through the mild acid hydrolysis of κ/β-CRG [23]. The κ/β-CRG (200 mg) was dissolved in 0.1 N hydrochloric acid (20 mL) and kept for 24 h at 37 °C; the depolymerization reaction was terminated by neutralization with 0.1 M NaOH. The obtained oligosaccharides were precipitated with five volumes of ethanol and centrifuged at 10.000 rpm^−1^ for 20 min at 4 °C. The pellet was collected, dissolved in water, dialyzed through a 1 kDa membrane, and lyophilized. These low-molecular-weight κ/β-CRG (LMW-κ/β-CRG)s were used for antiviral activity assays.

### 3.4. Enzymatic Degradation of the κ/β-CRG

The κ/β-CRG (KCl-insoluble fraction) was used for enzymatic degradation.

The enzymatic degradation was produced according to [10,11], using recombinant *Alteromonas carrageenovora* carrageenase. Prior to freeze-drying, the degradation products were filtered through Amicon Centriprep (YM-30 Millipore, Burlington, MA, USA) to obtain oligosaccharides and a resistant fraction of CRG, as described in [10]. The resistant fraction was designated as RF/CRG.

#### Degradation of RF/CRG (κ/β-CRG Resistant Fraction)

The mild acid hydrolysis of RF/CRG (κ/β-CRG was conducted by the method described by Yu et al. [23]. The RF/CRG (50 mg) was dissolved in 0.1 N hydrochloric acid (5 mL) and kept for 24 h at 37 °C. The depolymerization reaction was terminated by neutralization with 0.1 M NaOH. The obtained oligosaccharides were precipitated with five volumes of ethanol and centrifuged at 10.000 rpm^−1^ for 20 min at 4 °C. The pellet was collected, dissolved in water, dialyzed through a 1 kDa membrane, and lyophilized.

### 3.5. Instruments

All the MS experiments were performed using ultra-pure water, produced with Direct-Q 3 equipment (Millipore, Burlington, MA, USA). A MALDI matrix (2,5-dihydroxybenzoic acid, DHB) was purchased from Sigma (Burlington, MA, USA).

The MALDI-TOF mass spectra were recorded with an Ultra Flex III MALDI-TOF/TOF mass spectrometer (Bruker, Germany) with a SmartBeam laser (355 nm), a reflector, and potential LIFT tandem modes of operation. The instrument settings for the negative-ion mode were as follows: accelerating voltage, −25 kV; lase power, 19%; number of shots, 200; laser shot rate, 66 Hz. The sample preparation was as follows: a total of 2 μL of the mixture, containing 0.5 μL of the sample (5 mg/mL) in water, 1μL of DHB matrix (50 mg/mL) in 1:1 acetonitrile–water solution was mixed and introduced onto the sample plate and air dried.

The ESIMS/MS spectra were recorded with an ESI Q-TOF mass spectrometer (Agilent 6520 LC Q-TOF, Santa Clara, CA, USA) with a dual electrospray-ionization source. The instrument settings for the negative-ion ESIMS/MS were as follows: the spectra were acquired in the negative-ion mode with pre-calibration using a standard “HP-mix” for negative-ion mode. The capillary voltage was 4 kV; the drying gas temperature was 325 °C; and the fragmentor voltage was 160 V. The isolation window for MS/MS experiments was set to 1.3–3 mass units. The collision energy was optimized by fragmentation abundance. The dried sample was dissolved in 1:1 acetonitrile–water (the concentration of the sample was approximately 0.01 mg/mL) and introduced into the mass spectrometer at flow rate of 5 μL/min using a syringe pump (KD Scientific, Holliston, MA, USA).

The NMR spectra were obtained using an Avance DPX-500 NMR spectrometer (Bruker, Germany) resonating at 75.5 MHz at 35 °C. The concentration of the samples was 5–20 mg of polysaccharide/mL of D_2_O for 1D and 2D experiments.

The GLC–MS of alditol acetatederivatives were performed using a Hewlett-Packard 6850 (USA) chromatograph equipped with HP-5MS capillary column (30 m × 0.2 mm) with a temperature gradient of 150 → 230 °C at 3 °C/min.

The FTIR spectra of the polysaccharide (as films) was recorded on Invenio S and Equinox 55 Fourier transform spectrophotometers (Bruker, Germany), taking 120 scans with 4 cm^−1^ resolution. The OPUS/IR version 7.2 program set was used to measure the frequencies of absorption bands (AB) in IR spectra. The precision of the frequency measurements was ≤0.5 cm^−1^. The spectra regions of 1900–700 cm^−1^ were used, and the baseline was corrected for scattering. The spectra were normalized by the monosaccharide ring skeleton absorption at 1074 cm^−1^ (A_1074_ ≈ 1.0).

### 3.6. General Methods

#### 3.6.1. Analytical Procedures

The monosaccharide composition was determined through total reductive hydrolysis [27,28]. Neutral monosaccharides were analyzed as alditol [28] and aldononitrile [27] acetate derivatives by gas-liquid chromatography with a 6850 chromatograph (Agilent Technologies, Santa Clara, CA, USA) equipped with a capillary column HP-5MS (30 m × 0.25 mm, 5% Phenyl Methyl Siloxane) and a flame-ionization detector. The analyses were carried out using a temperature gradient program from 175 to 225 °C; the rate of temperature change was 3 °C/min. The sulfate ester content of polysaccharides was determined by the turbidimetric method [29].

#### 3.6.2. Molecular Weight Estimation

The viscosimetric molecular weights of the polysaccharide samples were calculated using the Mark–Houwink equation: [η] = KM^α^, where [η] is the intrinsic viscosity and *K* and *α* are empirical constants for κ-CRG constituting 3 × 10^−3^ and 0.95 at 25 °C in 0.1 M NaCl, respectively, according to the research data for this polymer–solvent system [30]. The viscosity of the CRG solutions (0.1–1.0 mg/mL in 0.1 M NaCl) was measured in a modified Ubbelohde viscometer (Pushino, Russia) with a capillary diameter of 0.3 mm at 25 °C, the time of accuracy being within ± 0.1 s. The intrinsic viscosity of the CRG samples was calculated through the extrapolation of the dependence ln (η)rel/C to infinite dilution using the least square method.

The MW of oligosaccharide samples was determined through the reducing sugars method with ferricyanide [31].

### 3.7. Antiviral Activity

#### 3.7.1. Cell Culture

The HEK293 (human embryonic kidney cells were cultured in a DMEM medium (Gibco/Invitrogen Life technologies) supplemented with 10% of fetal bovine serum (FBS), 4 mM of L-glutamine, 100 U/mL of penicillin, and 100 μg/mL of streptomycin. The Jurkat JMP (human T-lymphoblastic leukemia Clone 4 × 4#3) and Lan-1 (human neuroblastoma) were cultivated in RPMI-1640 containing 10% FBS, 4 mM of L-glutamine, 100 U/mL of penicillin, and 100 μg/mL of streptomycin. The cells were grown at 37 °C and 5% CO_2_ in a humid air atmosphere. The HEK293 and Lan-1 cells were kindly provided by Prof. Carol Stocking (University Medical Center Hamburg-Eppendorf, Hamburg, Germany); the Jurkat JMP were provided by Andreas Guse (University Medical Center Hamburg-Eppendorf, Hamburg, Germany).

#### 3.7.2. Cytotoxicity Assay

The cytotoxicity of the CRGs for the Jurkat JMP Lan-1 was determined by counting the number of viable cells using Trypan blue staining solution (Invitrogene Corp., Carlsbad, CA, USA). For this purpose, the CRGs were added to the medium in a concentration range between 0.01 µg/mL and 100 µg/mL. At 72 h post-treatment with CRGS, the Lan-1 cells were removed by trypsin, resuspended in medium, and stained with 0.4% Trypan blue solution for 5 min. The same manipulations, apart from the treatment with trypsin, were performed on the Jurkat JMP. Next, the number of viable (unstained) cells were counted in a Neubauer chamber.

#### 3.7.3. Obtaining of Pseudotyped Lentiviral Vectors Particles

The HEK293 packaging cells were seeded in 100 mm Petri dishes at 3 × 10^6^ cells per dish, 12–14 h before transfection. To obtain the Gp160, VSVG or McERV-pseudotyped RDLVs, we used 10 µg of lentiviral LeGO plasmid encoding marker eGFP gene) PMID: 18362927, PMID: 26020616), 10 µg of gag-pol plasmid, 5 µg of Rev plasmid, and 2 µg VSV-G; a total of 4 µg Gp160 or 5 µg McERV envelope protein encoding vector were introduced into the HEK293 cells via calcium phosphate transfection (ProFection^®^ Mammalian Transfection System, Promega). At 8 h after transfection, the medium was changed with DMEM containing 20 mM HEPES. After 24 h, the supernatants containing GP160, VSVG- or McERV-pseudotyped viral particles were collected, filtered through 0.22 mm filter (Millipore) and stored at −80 °C.

#### 3.7.4. Antiviral Activity Assay

The antiviral activity of the sulfated polysaccharides was studied using the Jurkat JMP and Lan-1 cell lines. GP160/McERV/VSVG pseudotyped lentiviral vectors particles were used for transduction. For the transduction, the Jurkat JMP were seeded at 5 × 10^4^ per well of 24 well plate. The Lan-1 cells were seeded at 2 × 10^4^ cells per well. One hour before transduction the carrageenans were added at final concentrations of 0.1, 1.0, 5.0, 10.0, and 25.0 μg/mL. In this study, the same titers (5 × 10^5^–5 × 10^6^ units/mL) of lentiviral vector particles were used. The number of fluorescent cells was counted by a Flow cytometer BD LSRFortessa 72 h post-transduction with the pseudotyped lentiviral vectors. FlowJo X software (Tree Star Inc., Ashland, OR, USA) was used for the data analysis.

#### 3.7.5. Statistical Analysis

All the data are presented as mean ± SD. The statistical significance of the differences observed in the cell count and in the pseudotyped lentiviral vector particle transduction efficiency experiments was determined by the Mann–Whitney non-parametric test. The difference was considered statistically significant at *p* < 0.05. All the statistical calculations were performed using GraphPad Prism 8 software.

## 4. Conclusions

New insights into the structure of the hybrid κ/β-CRG) of the red alga *T. crinitus* were obtained. For this purpose, CRG oligosaccharides were prepared through chemical and enzymatic depolymerization and characterized using laser desorption ionization with a negative ion matrix (MALDI-TOFMS) and electrospray ionization mass spectrometry. A mass spectrometric approach in the investigation of the composition and distribution of the repeating units of κ/β-CRG enlightened the structural features of this complex hybrid polysaccharide. It was found that the composition of oligosaccharides derived from a resistant fraction by mild acid hydrolysis, despite partial desulphation, was characterized by blocks, which were inaccessible for cleavage by the enzyme. The fragments contained κ-, β-, and ι-inserts, and μ- and ν-inserts. The precursor molecules to the unsulphated β-units were also found: the positive-ion ESIMS of the RF/CRG hydrolysis mixture revealed the presence of the ion series of (G +Na), (G-D +Na)^+^ and (G-D-G +Na)^+^. These blocks were the residues of unsulphated, extensive chains built up of β-type units. The information obtained on the structural features of RF/CRG explains the stability of the part of κ/β-CRG against enzymatic hydrolysis by κ-carrageenase. Thus, preparing κ/β-CRG derived oligosaccharides through chemical and enzymatic depolymerization and investigating them using FT-IR and NMR spectroscopy and mass spectrometry allowed us to prove and to characterize the hybrid structure of this polysaccharide.

As demonstrated by the results, the antiviral activity of the investigated CRGs depends on the type of virus and, more precisely, on the envelope protein species. The polysaccharide κ/β-CRG and its oligosaccharide significantly inhibit the infection of cells with replication-defective viruses pseudotyped with envelope proteins GP160 and McERV, and exert a less pronounced antiviral effect on the virus pseudotyped with VSVG, which was intrinsic to both the cell lines used in this study. These results suggest that CRGs possibly act more selectively against certain types of viruses. For example, GP160 and McERV are retroviral envelope proteins, but the VSVG protein is a coating protein of vesicular stomatitis virus, which belongs to another family of rhabdoviruses. This point needs further investigation because very restricted amounts of various viral envelope proteins were used in our study. Importantly, the investigated polysaccharide and its oligosaccharide dramatically reduced the transduction efficiency of replication-defective HIV-1 particles containing GP160—the envelope protein of the human immunodeficiency virus HIV-1—when added to T-lymphocyte Jurkat cells. The CRG oligosaccharide displayed significantly higher antiviral activity.

Here, for the first time, we provide evidence that original κ/β-CRGs and their oligosaccharides offer pronounced antiviral potential in non-toxic doses and deserve further in vitro and in vivo investigations to evaluate their potential for possible therapeutic applications as antiviral agents.

## Figures and Tables

**Table 1:** Chemical composition of κ/β-carrageenans.

Source	Sample of CRGs	Composition, Dry Weight %			MW, kDa
		Gal	3,6 AnGal	SO_4_^−2^	
CRG-T. crinitus	κ/β-	39.2	33.5	20.8	350.0
*Oligosaccharides (LMW)*	κ/β-	38.1	33.0	21.0	2.1

**Table 2:** Structural features of some oligosaccharides, obtained by mild acid hydrolysis of high-molecular-weight RF/CRG fraction, which was not hydrolyzed by κ-carrageenase from marine bacterium *P. carrageenovora.*

*m*/*z*		Composition and Structural Features of Oligosaccharides
MALDI	ESI	
259.0	259.004	G4S
403.1	403.034	G4S-DA
565.1	565.079	G4S-DA-G, G-DA-G4S
583.1	583.089	G4S-D-G, G-D6S-G
667.0	322.015	G4S-DA-G4S, G-DA2S-G4S
685.1	331.018	G4S-D6S-G,G-D6S-G4S
709.1	-	fragment
727.0	-	fragment
811.0	394.041	(G4S-DA)_2_
829.1	403	G4S-DA-G4S-G
871.2	871.188	G4S-DA-G-DA-G, G-DA-G4S-DA-G, G-DA-G-DA-G4S
889.3	-	fragment
1009.1	-	fragment from 355.023^3-^
1033.2	-	fragment from 295.010^3-^
1177.4	277.005	G4S-DA2S-G4S-DA2S-G
1315.3	646.106	G4S-D-G4S-D-G-DA
1483.4	-	fragment
1585.4	-	fragment
1789.6	-	fragment
1891.5	-	fragment
2095.7	-	fragment

## Data Availability

https://www.mdpi.com/ethics (accessed on 24 November 2021).

## Supplementary Materials

The following are available online at https://www.mdpi.com/article/10.3390/ijms222312905/s1.
