# Supplementary material for: New Insights into the Structure of Kappa/Beta-Carrageenan: A Novel Potential Inhibitor of HIV-1
Source: Int J Mol Sci. 2021 Nov 29;22(23):12905. doi: 10.3390/ijms222312905 (PMC8657973; doi:10.3390/ijms222312905)
Supplement: Supplementary file 1 [file ijms-22-12905-s001.zip › ijms-1460541-supplementary.pdf]

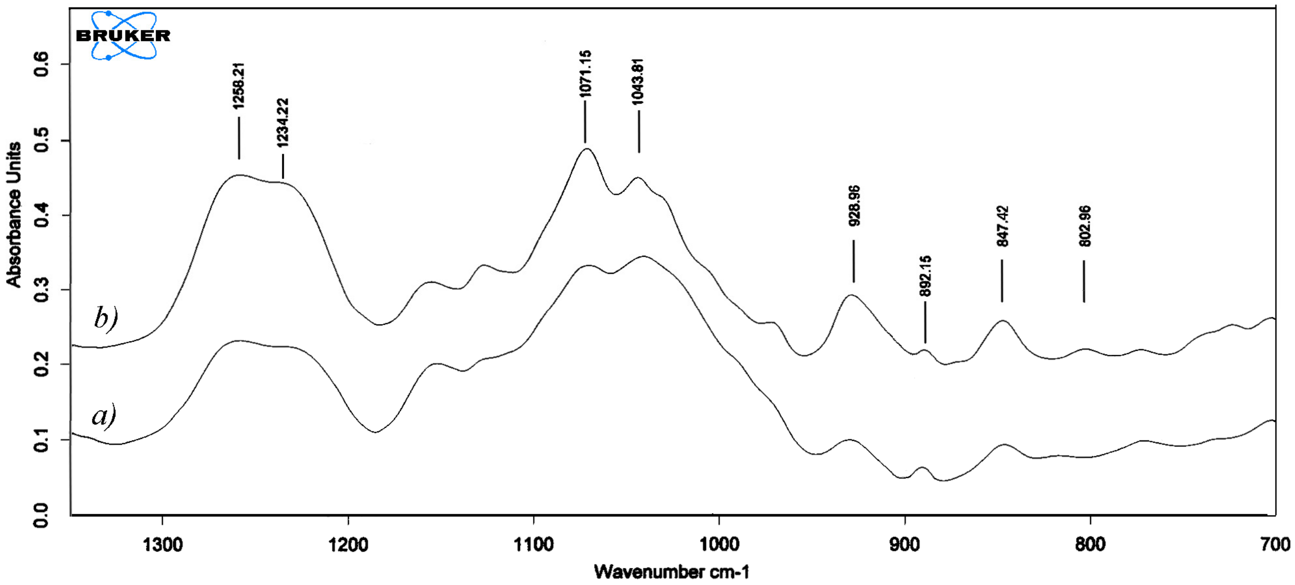

Supplementary S1. The FT-IR spectra of the a) κ/β-CRG and b) LMW–CRG.

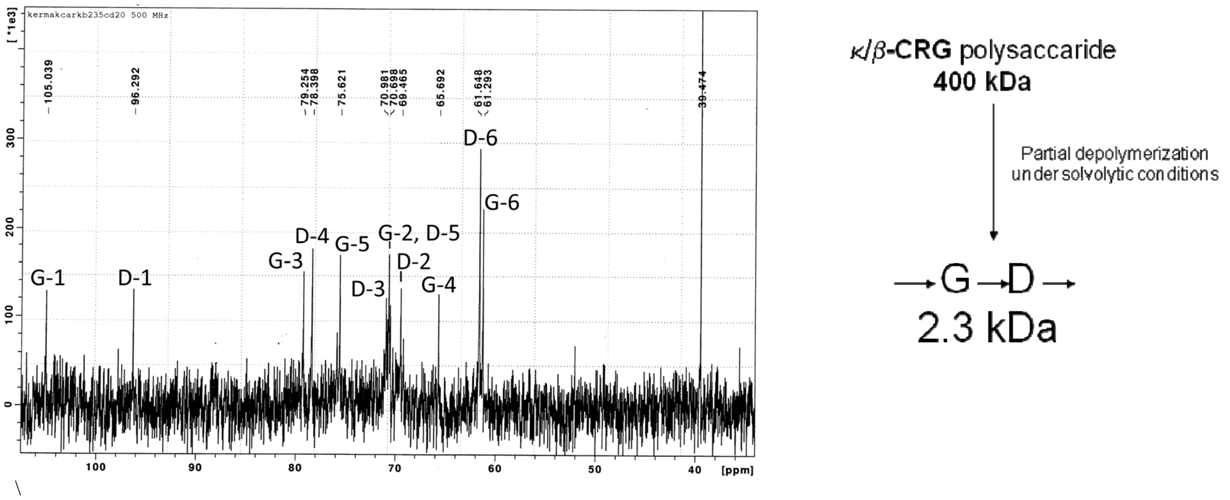

Supplementary S2. ^{13}C NMR of solvolytic desulfation/degradation products, obtained from ***κ/β*** **-CRG.**

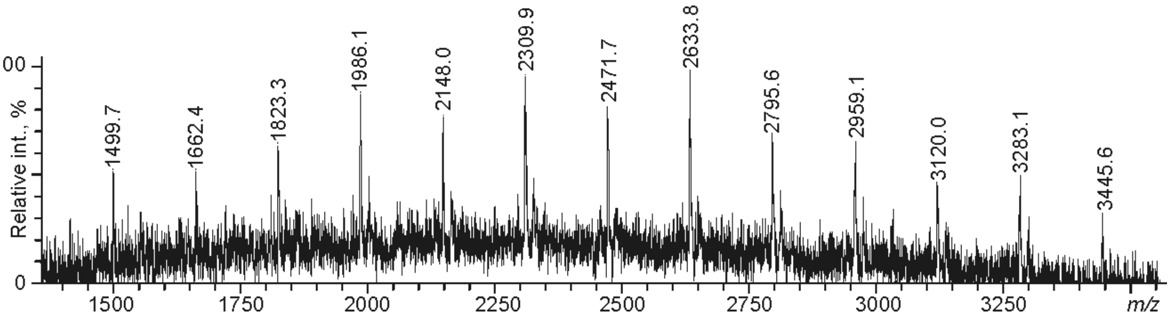

Supplementary S3. Positive-ion MALDI-TOFMS of polysaccharide, obtained from ***κ/β*** **-CRG** by solvolytic desulfation/degradation.
